# Testing for neural antibodies in autoimmune encephalitis: who, what, where, when, why, and how

**DOI:** 10.1177/17562864261429181

**Published:** 2026-03-12

**Authors:** Shaza Almweisheer, Kamala Sangam, Ola Ismail, Adrian Budhram

**Affiliations:** Department of Clinical Neurosciences, University of Calgary, Calgary, AB, Canada; National Neuroscience Institute, King Fahad Medical City, Riyadh, Saudi Arabia; Department of Clinical Neurological Sciences, London Health Sciences Centre, Western University, London, ON, Canada; Department of Pathology and Laboratory Medicine, London Health Sciences Centre, Western University, London, ON, Canada; Department of Clinical Neurological Sciences, London Health Sciences Centre, Western University, 339 Windermere Rd, London, ON N6A 5A5, Canada; Department of Pathology and Laboratory Medicine, London Health Sciences Centre, Western University, London, ON, Canada

**Keywords:** autoantibody testing, autoimmune encephalitis, neural antibody testing, neural antibody-associated disease

## Abstract

In recent decades, neural antibody testing has emerged as a cornerstone of autoimmune encephalitis (AE) diagnosis. Commercially available assays to detect these antibodies are now in widespread use, resulting in dramatically increased test accessibility and reduced turnaround times. However, there remains heterogeneity in testing approach and pitfalls related to test ordering as well as diagnostic test performance, which may vary across laboratories depending on their testing methodologies or algorithms. This creates the potential for both false-negative and false-positive results, which can lead to patient misdiagnosis if clinicians are not familiar with both the strengths and limitations of this testing in practice. In this narrative review, we discuss the approach to patient selection for antibody testing, importance of serum and cerebrospinal fluid testing using appropriately handled specimens, potential limitations of local versus reference laboratory testing, optimal timing of testing, benefit of testing to patient management, and diagnostic performance of different test methodologies and algorithms ( the “who, what, where, when, why, and how” of neural antibody testing in AE), with the aim of providing a practically useful framework for clinicians who order this testing.

## Introduction

Despite the central importance of testing for neural antibodies (i.e., antibodies against neuronal or glial antigens) to the diagnosis and management of autoimmune encephalitis (AE), there remains heterogeneity in testing approach and pitfalls related to test ordering and interpretation.^1–4^ In recent years, an increasing number of laboratories worldwide have begun offering neural antibody testing for AE using commercially available assays, resulting in greater test accessibility and shorter turnaround times. However, diagnostic test performance can vary across laboratories that implement different test methodologies or algorithms to detect these antibodies, creating the potential for both false-negative and false-positive results.^5–10^ In this narrative review, we discuss the approach to patient selection for antibody testing, importance of serum and cerebrospinal fluid (CSF) testing using appropriately handled specimens, potential limitations of local versus reference laboratory testing, optimal timing of testing, benefit of testing to patient management, and diagnostic performance of different test methodologies and algorithms ( the “who, what, where, when, why, and how” of neural antibody testing in AE), with the aim of providing a practically useful framework for clinicians who order this testing.

## Neural antibodies in AE: Extracellular versus intracellular targets

Neural antibodies can be classified by whether their targets are extracellular or intracellular, which is often relevant to their method of detection in clinical laboratory practice. Antibodies against extracellular targets typically bind cell-surface or synaptic proteins and are thought to be pathogenic, causing dysfunction through various mechanisms including crosslinking and internalization, direct functional blockade, disruption of protein–protein interactions, and complement activation.^11–13^ Because detection of antibodies against extracellular targets generally requires that the antigen be in its conformational state, cell-based assays (CBAs) that express native conformational antigen are often used to test for them (discussed later).^1,14,15^ In contrast to antibodies against extracellular targets, those against intracellular targets are generally not thought to be directly pathogenic but instead surrogate markers of cytotoxic T-cell mediated pathology.^16^ Detection of these antibodies typically does not require that the antigen be in its native conformational state, so assays such as immunoblot (IB) are frequently used for their detection (discussed later).^1,15,17^ An in-depth review of the clinical management of patients with AE and antibodies against extracellular versus intracellular targets, which can inform choice of immunotherapy, cancer screening, and prognostication, is beyond the scope of this manuscript but is discussed elsewhere.^18–21^

## Neural antibodies in AE: Why and where to test

Testing for neural antibodies in patients with suspected AE has numerous practical benefits. In patients who lack prompt response to these first-line immunotherapies (i.e., corticosteroids and intravenous immunoglobulin (IVIG) or plasma exchange (PLEX)), making the decision to escalate to a second-line immunotherapy that may more intensively or protractedly suppress the immune system (e.g., rituximab or cyclophosphamide) is often aided by the greater diagnostic certainty that is provided by a highly specific antibody result.^19^ Furthermore, knowledge of the antigenic target (e.g., intracellular vs extracellular) can shed light on preferred second-line immunotherapy, with B-cell depleting agents such as rituximab thought to potentially be more beneficial in patients with disease mediated by pathogenic antibodies against extracellular targets.^19^ In some regions, a greater degree of diagnostic certainty may be required to obtain access to more costly second- or third-line immunotherapies, including monoclonal antibodies such as rituximab or tocilizumab. A positive antibody result can also aid tremendously in guiding screening for occult tumor, and facilitate access to costly imaging modalities that more sensitively detect cancer such as PET.^19,22,23^ Identifying a particular neural antibody additionally helps inform disease prognosis, with rapidly evolving literature describing antibody-specific long-term patient outcomes.^24^ Finally, with a number of clinical trials underway for patients with antibody-mediated encephalitides, it is plausible that a positive antibody result will be required to access these treatments should they prove to be effective.^25,26^

While neural antibody testing should generally be performed in an accredited laboratory to ensure quality and competence through conformance to an acknowledged standard, the specific laboratory selected for testing is often dictated by institutional policies and regional limitations.^27,28^ Practically speaking, however, clinicians still benefit from being familiar with strengths and limitations of different testing methodologies (discussed later), knowledge of which is helpful when advocating for change to local testing approaches or algorithms that can improve diagnostic accuracy. For laboratories that are unable to implement certain assays that are central to the diagnostic evaluation of AE (e.g., tissue-based assay (TBA), discussed later), collaboration between the clinician and laboratorian to develop algorithms for send-out testing to reference laboratories in patients with concern for false-positive or false-negative results generated locally can be helpful.^2,19^ For patients without identifiable antibodies following comprehensive evaluation by clinical service basis assays, reference laboratories may also be able to use cultured live neurons to detect antibodies against unknown neuronal surface antigens^29,30^; while this testing can be of value in persistently suspicious cases with otherwise negative exhaustive investigations, it is primarily performed on a research basis at this time and is thus not discussed further. Given the variability in testing offered across laboratories, (e.g., variable assays used for antibody detection, or breadth of assays incorporated in antibody panels), open communication with the testing laboratory is strongly recommended for the clinician to better understand how their patient antibody results are generated and practically apply the concepts discussed herein. Finally, it is important to remember that neural antibody discovery has progressed rapidly over the last decade and continues to do so; for this reason, in patients with a high index of suspicion for AE who are antibody-negative by locally available testing, consultation with an expert in autoimmune neurology to review the potential benefit of any further testing (e.g., send-out testing, research basis testing) is suggested.

## Neural antibodies in AE: Who to test

Suspicion for AE is generally high in patients with the syndrome of encephalitis and no clear etiology identified following expert obtainment and review of the clinical history, physical examination, and results of routine general medical and neurologic investigations (e.g., brain magnetic resonance imaging (MRI), CSF viral testing). Such patients classically present with subacute (<3 months) neuropsychiatric symptoms along with supportive evidence of neuroinflammation (e.g., on MRI or CSF profile), and are well-captured by the criteria for possible AE.^31^ Notably, however, some subtypes of AE may present relatively pauci-symptomatically (e.g., with seizures in relative isolation) or more insidiously (e.g., >3 months), including AE associated with anti-leucine-rich glioma-inactivated 1 (LGI1), anti-contactin-associated protein-like 2 (CASPR2), antidipeptidyl-peptidase-like protein 6 (DPPX), or/and anti-IgLON family member 5 (IgLON5).^32–37^ Recognition of characteristic clinical features associated with these antibodies (e.g., faciobrachial dystonic seizures associated with anti-LGI1) is thus critical to pursuing testing in patients who do not meet possible AE criteria.^37^ A comprehensive summary of clinical presentations observed with individual forms of neural antibody-associated AE can be found elsewhere,^38^ but because of substantial syndromic overlap it is generally recommended that comprehensive panel-based neural antibody testing (discussed later) be performed in patients with suspected AE to avoid diagnostic delays.^1,19^ Timely confirmation of AE diagnosis can help facilitate earlier immunotherapy, which has been associated with better prognosis in AE.^39–41^

Depending on the patient presentation, predictive scores have been proposed to help select those who would benefit from neural antibody testing to diagnose AE. Among patients with seizures of unknown etiology, examples include the Antibody Prevalence in Epilepsy and Encephalopathy (APE2) score, the Oxford Autoimmune Neurology Group score, the Antibodies Contributing to Focal Epilepsy Signs and Symptoms (ACES) score, and combinations such as the APE2/ACES reflex score.^30,42–44^ The performance of these scores is dependent on the population in which they are applied; as examples, the APE2 score is considered more useful in patients with subacute onset of seizures along with other features suggestive of encephalitis, while the ACES score is considered more useful in patients with longer duration of seizures in relative isolation.^42–44^ While predictive scores have been found to perform reasonably well in initial validation studies, their diagnostic advantage when compared to only ordering neural antibody testing in patients with “obvious” indications as recognized by the clinician remains unclear. The ACES study attempted to address this by only including patients who had no features to suggest an autoimmune etiology that were recognized by the referring clinician.^43^ This resulted in an expectedly low number of patients with antibody positivity in this study (3.4%); however, it was identified in retrospect that virtually all these patients had features that were characteristic of neural antibody-associated presentations but went unrecognized by the referring clinician.^43^ The “Obvious” indications for neural antibody testing in epilepsy or seizures (ONES) checklist, which consists of presentations that are individually suspicious for neural antibody positivity, was developed to operationalize the reference standard of clinician recognition against which predictive scores could be compared.^45^ Studies comparing predictive scores to the ONES checklist have not reproducibly found them to confer a diagnostic advantage, casting doubt on their role in clinical practice and suggesting there may be greater value in clinician education to improve recognition of presentations suspicious for neural antibody-associated disease.^45,46^

We recommend promptly pursuing neural antibody testing in all patients with the syndrome of encephalitis who meet possible AE criteria.^31^ For patients with more chronic or fragmentary presentations, such as seizures or brainstem dysfunction in relative isolation, recognition of clinical features or ancillary test data that are both characteristic of neural antibody-associated disease and not attributable to a more likely diagnosis is essential to flagging the need for antibody testing.^37^ Because such patient presentations may span the full spectrum of neurologic subspecialities (e.g., epilepsy, movement disorders, cognitive neurology, and neuromuscular disorders), subspecialist review of competing diagnostic considerations (e.g., functional, degenerative, toxic/metabolic, genetic) along with interdisciplinary discussion that includes autoimmune neurology input can be invaluable to identifying those who do not meet possible AE criteria but may benefit from neural antibody testing.

## Neural antibodies in AE: How to test

Numerous assays are in use to detect neural antibodies, including TBA, IB, CBA, enzyme-linked immunosorbent assays (ELISA), and radioimmunoprecipitation assays (RIA). A summary of these assays as they pertain to neural antibody testing in AE is provided in Table 1.^19^ Misinterpretation of antibody results has been found to be a major contributor to patient misdiagnosis^47^; a basic understanding of the assays in use for neural antibody detection, including their strengths and limitations, is therefore necessary for the clinician ordering this testing to avoid diagnostic pitfalls. While beyond the scope of this manuscript, more technical descriptions of assays with schematic representations can be found elsewhere.^15^

**Table 1.** Test methodologies employed for neural antibody detection in patients with suspected AE.

Method	Examples of antibodies detected	Comments
TBA	Various	Requires expertise in interpretation of neural antibody tissue staining patterns Useful to screen for neural antibodies against intracellular and extracellular antigens, although sensitivity of commercial TBA reported to be suboptimal Can be used to corroborate positive IB or CBA results to maximize specificity Does not reliably detect certain antibodies (e.g., anti-MOG, GlyR, recoverin, titin)
CBA	Anti-NMDAR, LGI1, CASPR2, GABA(B)R, AMPAR, DPPX, GAD65, IGLON5, MOG, GLYR	CBA reported to have higher sensitivity than TBA for certain neural antibodies (e.g., anti-LGI1, CASPR2); however, higher sensitivity may come at cost to specificity Specificity of isolated positivity by CBA varies across analytes and is lower in the absence of corresponding positivity by second assay (e.g., TBA); for weak/low isolated serum positivity by CBA, discuss further evaluation with testing laboratory (e.g., dilutions to determine endpoint titer for anti-CASPR2) Note that CBAs for anti-MOG and anti-GlyR are not routinely incorporated in neural antibody panels for AE, but should be ordered in patients with compatible disease phenotypes (e.g., ADEM or CCE/FLAMES for anti-MOG, PERM for anti-GlyR); restricting testing of these antibodies to patients with compatible disease phenotypes reduces proportion of false-positives, which usually occur as low levels of positivity in serum
IB	Anti-Hu, Yo, Ri, amphiphysin, CV2/CRMP5, Ma2/Ta, SOX1, Zic4, Tr/DNER, GAD65	Specificity of isolated positivity by IB varies across analytes and is lower in the absence of corresponding positivity by second assay (e.g., TBA)
RIA	Anti-GAD65 Anti-VGKC	Serum cut-offs for what constitutes high levels of anti-GAD65 by RIA have been published (>20 nmol/L or >2000 U/mL); in cases of diagnostic uncertainty, testing CSF alongside serum to assess for intrathecal synthesis of anti-GAD65 has been suggested but more evidence is needed Detection of anti-VGKC in the absence of anti-LGI1/CASPR2 lacks specificity for neurologic autoimmunity; testing for anti-VGKC is not recommended
ELISA	Anti-GAD65	Serum and CSF cut-offs for what constitutes high levels of anti-GAD65 by ELISA have been published (>10,000 IU/mL for serum, >100 IU/mL for CSF); in cases of diagnostic uncertainty, testing CSF alongside serum to assess for intrathecal synthesis of anti-GAD65 has been suggested but more evidence is needed

Source: Table adapted and re-used from Cambridge University Press: Hahn et al.^19^ This is an open access article distributed under the terms of the Creative Commons CC BY license, which permits unrestricted use, distribution, and reproduction in any medium, provided the original work is properly cited.

ADEM, acute disseminated encephalomyelitis; AE, autoimmune encephalitis; AMPAR, α-amino-3-hydroxy-5-methyl-4-isoxazolepropionic acid receptor; CASPR2, contactin-associated protein-like 2; CBA, cell-based assays; CCE, cerebral cortical encephalitis; CRMP5, collapsin response-mediator protein-5; CSF, cerebrospinal fluid; DNER, delta/Notch-like epidermal growth factor-related receptor; DPPX, dipeptidyl-peptidase-like protein 6; ELISA, enzyme-linked immunosorbent assays; FLAMES, FLAIR-hyperintense lesions in anti-MOG-associated encephalitis with seizures; GABA(B)R, γ-Aminobutyric acid type B receptor; GAD65, glutamic acid decarboxylase-65; GlyR, glycine receptor; IB, immunoblots; IgLON5, IgLON family member 5; LGI1, leucine-rich glioma-inactivated 1; MOG, myelin oligodendrocyte glycoprotein; NMDAR, N-methyl-D-aspartate receptor; PERM, progressive encephalomyelitis with rigidity and myoclonus; RIA, radioimmunoassays; SOX1, SRY-box transcription factor 1; TBA, tissue-based assays; VGKC, voltage-gated potassium channel.

### Tissue-based assays

**Figure 1.**
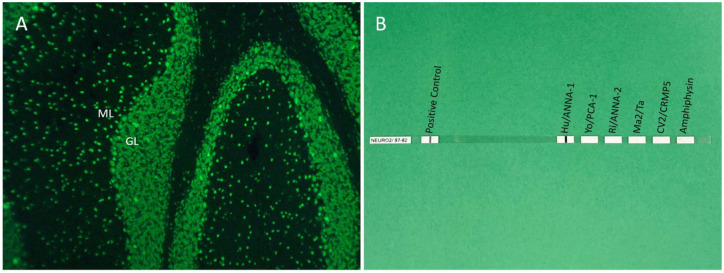
Positivity for anti-Hu/ANNA-1 by mouse brain TIIF and commercial LB. Mouse brain TIIF shows cerebellar staining typical of anti-Hu/ANNA-1 (a), alongside positivity for anti-Hu/ANNA-1 by commercial LB (b). Source: Figure reused from Budhram and Flanagan,^48^ with permission from Elsevier. GL, granular layer of the cerebellum; LB, line blot; ML, molecular layer of the cerebellum; TIIF, tissue indirect immunofluorescence.

**Figure 2.**
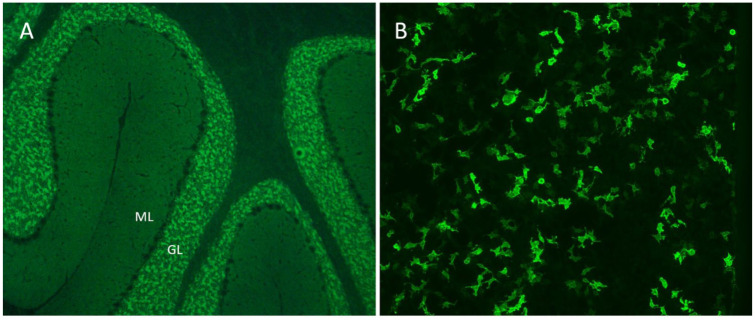
Positivity for anti-DPPX by mouse brain TIIF and commercial fixed CBA. Mouse brain TIIF shows cerebellar staining typical of anti-DPPX (a), alongside positivity for anti-DPPX by commercial fixed CBA (b). Source: Figure reused from Budhram and Flanagan,^48^ with permission from Elsevier. CBA, cell-based assay; DPPX, dipeptidyl-peptidase-like protein 6; GL, granular layer of the cerebellum; ML, molecular layer of the cerebellum; TIIF, tissue indirect immunofluorescence.

The use of TBA, which includes immunohistochemistry and tissue indirect immunofluorescence (TIIF), allows laboratories to reduce their risk of false-negatives by comprehensively screening for a breadth of antibodies against intracellular and extracellular targets.^1,15^ It also aids in confirming positivity observed by monospecific assays such as CBA or IB because, with few exceptions (e.g., antiglycine receptor (GlyR), antimyelin oligodendrocyte glycoprotein (MOG)), true-positives would be expected to demonstrate corresponding positivity by TBA.^1,48^ In these ways, incorporation of TBA into testing algorithms for neural antibody detection in AE optimizes sensitivity and specificity. Sections from rat, mouse, or primate tissues are typically used for TBA, including brain tissue components (e.g., hippocampus, cortex, cerebellum, thalamus, basal ganglia, brainstem) as well as nonbrain tissues (e.g., gut mucosa, kidney).^1,15^ Patient serum or CSF is incubated with these sections. With TIIF, an antihuman IgG secondary antibody conjugated to a fluorescent dye is used to detect tissue-bound antibodies.^1,15^ Using microscopy, assay positivity is determined by visualization of characteristic neural-specific staining patterns, with inclusion of nonbrain tissues serving as nonneural controls. In patients with staining suggestive of a particular neural antibody, reflex confirmatory testing using a monospecific assay such as IB (see Figure 1) or CBA (see Figure 2) is performed; diagnostic confirmation by two assays indicates a highly specific antibody result.^10,48^ This was exemplified by a retrospective study of 96 samples that were positive for high-risk paraneoplastic antibodies by commercial LB and evaluated by TBA.^7^ Of those positive by LB and TBA (concordant group, 46/96 (48%)), 91% were classified as true-positives, while of those positive by LB alone (discordant group, 50/96 (52%)), only 8% were classified as true-positives.^7,8^ While much of the early work describing use of TBA to sensitively screen for neural antibodies was based on in-house TBAs performed at reference laboratories, commercial TBAs are now available and in widespread use. Recent evaluations of commercial TBAs have reported suboptimal overall sensitivity of 65%–75%, with variation by manufacturer as well as antigenic target.^8,9^ In laboratories reliant on commercial TBA, testing alongside more sensitive monospecific commercial assays (e.g., commercial LB and CBA) is therefore recommended, and retesting at reference laboratories using in-house TBA should be considered in cases with discordant results if there is persistent diagnostic uncertainty.^8,9^ Observation of neural-specific staining on TBA (i.e., clear staining of brain tissue components but not nonbrain tissues) that is not recognizable may be indicative of an uncharacterized neural-specific antibody; such findings may be supportive of neurologic autoimmunity in the appropriate clinical context, even if the antigenic target is not determinable outside of research settings.^49^ However, clinical correlation is particularly important in such cases because uncharacterized antibodies may also occur in patients with alternative diagnoses (e.g., acute CNS infection), possibly as an epiphenomenon.^50^ While it is a powerful diagnostic tool, implementation of TBA can be costly, labor-intensive, and requires expertise in interpretation.^1,19^ For these reasons, laboratories may opt not to use TBA and rely solely on commercially available monospecific assays (e.g., IB or CBA) for neural antibody testing, which increases the risk of false-positive and false-negative results.^10,19,51^ Clinicians are therefore encouraged to discuss testing algorithms with their local laboratory in cases of diagnostic uncertainty, and review whether use of TBA may aid in reconciling unexpected neural antibody results.

### Immunoblots

The use of IB, including Western blot (WB) and LB, facilitates the detection of neural antibodies against intracellular targets. Historically, WB was more commonly used for neural antibody detection.^1,15^ Yet, WB may be generated from tissue extracts that contain thousands of proteins, which can lead to background staining that adversely impacts assay interpretation.^1,15^ More recently, the development of LB has allowed for specific antibody detection with reduced background staining. LB uses purified antigen that is transferred to a particular location on a membrane, which allows for multianalyte analysis by incubating patient sample with a single strip that is coated with multiple antigens at different locations.^1,15^ If an antibody of interest is present in the tested sample, it binds to the specific antigen on the strip and is detected by use of an enzyme-linked anti-human IgG secondary antibody.^1,15^ Commercially available LB kits are now in widespread use; however, detection of neural antibodies by LB alone, without demonstrating confirmatory positivity by TBA, has substantial limitations. This is best highlighted by studies of test positive predictive value (PPV), which refers to the probability that a patient with a positive test result truly has the disease of interest.^10^ The PPV of isolated LB positivity has reproducibly been shown to be poor at <50%, indicating a high proportion of false-positives when LB is used without TBA in clinical practice.^5,6,52^ For this reason, clinicians should review positive LB results with the testing laboratory to confirm whether corresponding positivity by TBA was observed, particularly in patients with atypical disease phenotypes.^2^ Positive IB results without corresponding positivity by in-house TBA performed at reference laboratories may uncommonly be found in patients who have a compatible malignancy; however, cancers detected in patients with isolated LB positivity are often not typical of the identified antibody, further underscoring the need for skepticism in patients with such antibody results.^7,53^ Interpretation of positive antibodies included in commercial LB for which TBA staining is not reliably observed when using brain tissue (e.g., anti-recoverin, anti-titin) can be especially challenging due to the lack of a confirmatory second assay, and in such patients clinical correlation is particularly important.^54^

### Cell-based assays

In clinical practice, CBA is primarily used to detect antibodies against extracellular targets. Most commonly, cells are transfected to express native conformational antigen, which can then bind the antibody of interest.^1,15^ With fixed CBA, the cells are chemically fixed to enable transportation and commercialization, which has dramatically increased accessibility of this test methodology worldwide. Fixation may however reduce sensitivity of testing for some antibodies.^55^ This has been highlighted with anti-MOG, for which sensitivity of fixed CBA has reproducibly been shown to be 10%–15% lower than that of live CBAs performed at specialized centers.^55–57^ In patients with a high index of suspicion for MOG antibody-associated disease but negative fixed CBA testing, arranging for live CBA testing at a specialized center should therefore be considered.^57^ Notably, however, for some antibodies, live CBA has been reported to be less specific than fixed CBA, such as when testing serum for anti-N-methyl-D-aspartate receptor (NMDAR); for this reason, comparative performance of live versus fixed CBA for a given antibody benefits from systematic evaluation of both sensitivity and specificity.^58,59^ With respect to antibody detection, cells are incubated with patient serum and a fluorescently labeled antihuman IgG antibody is subsequently used, allowing for visual determination of antibody positivity by microscope examination.^1,15^ There is an element of subjectivity to this visual assessment, which may contribute to false-positives if interpretation is performed by less experienced readers.^60^ This can be mitigated by use of quantitative flow cytometry, which similarly uses transfected cells in solution to measure antibody binding quantitatively.^15^ Nonetheless, false-positives remain possible for antibodies detected by all forms of CBA, including those reliant on visual interpretation or quantitative flow cytometry. For many antibodies associated with AE that are detected by CBA, corresponding positivity by TBA confirms a highly specific result. For certain antibodies that do not reliably stain tissue such as serum anti-MOG, knowledge of titers can be immensely helpful in informing specificity of a positive result; this is also true of certain neural antibodies for which characteristic staining patterns on TBA have been described but may not universally be present (e.g., serum anti-CASPR2 or anti-gamma-aminobutyric acid type B receptor).^28,61–66^ Anti-GlyR also does not reliably stain tissue and the false-positive rate of serum CBA testing for this antibody is reported to be as high as 4%, emphasizing the importance of clinical correlation to ensure a compatible disease phenotype (e.g., progressive encephalomyelitis with rigidity and myoclonus, or PERM) in any patient with a positive serum CBA result.^67,68^ Modification of GlyR CBA to detect serum antibodies causing antigen endocytosis (modulating assay) has been shown to improve specificity and, while not routinely clinically available, may be diagnostically helpful in select cases.^67^ When faced with diagnostic uncertainty relating to CBA results, clinicians are encouraged to contact the testing laboratory in order to discuss elements of testing such as visual appearance if applicable, whether corresponding positivity by TBA is present, and titers as well as any specialized testing that may be of particular value to interpretation of certain antibodies.

### ELISA and RIA

ELISA and RIA are two different widely used methodologies in clinical laboratories, which are grouped together here because their use in AE primarily relates to detection of anti-glutamic acid decarboxylase-65 (GAD65). Of historical note, RIA is also used to detect anti-voltage-gated potassium channel (VGKC), which was previously thought to define a subset of patients with AE; however, it was later determined that the specific antigenic targets in these patients were anti-LGI1/CASPR2, while anti-VGKC positivity in the absence of anti-LGI1/CASPR2 was found to lack disease specificity.^69^ For this reason, testing for anti-VGKC is not recommended and is not discussed further. Both RIA and ELISA have been used to distinguish low levels of anti-GAD65 (which may be found in patients with systemic autoimmune disease like type 1 diabetes mellitus (T1DM) and even healthy people), from high levels of anti-GAD65 (which have been associated with GAD65 neurologic autoimmunity).^70,71^ Antibody reactivity against the catalytic domain of GAD65 is typically demonstrable in patients with GAD65 neurologic autoimmunity, but does not distinguish among core disease phenotypes that include stiff person spectrum disorder (SPSD), cerebellar ataxia, limbic encephalitis, and temporal lobe epilepsy.^72,73^ When using RIA for antibody detection, the antigen is radiolabeled and incubated with patient sample followed by an anti-human IgG antibody that results in radioactive complexes when the antibody of interest is present.^1,15^ These complexes are then precipitated and their radioactivity is measured quantitatively.^1,15^ With respect to anti-GAD65, published cut-offs used in neurologic disease characterization studies to define “high levels” of anti-GAD65 in serum by RIA include 20 nmol/L and 2000 U/mL.^74,75^

More recently, ELISA has emerged as an attractive alternative to detect high levels of anti-GAD65 without the requirement for radioactive reagents. With ELISA, patient sample is most commonly incubated with antigen-coated wells, and the presence of the antibody of interest is detected with an anti-human IgG secondary antibody conjugated to an enzyme that, following substrate conversion, results in a color change that can be measured quantitatively.^15^ Variations in ELISA, including the use of antibody-coated wells or two antigen layers, are also employed clinically.^15^ “High levels” of anti-GAD65 by ELISA have been defined as >10,000 IU/mL in serum and >100 IU/mL in CSF.^76^ When assessing a patient with a “positive” anti-GAD65 result by RIA or ELISA, it is critical to clarify whether the patient has low levels (which lack specificity for neurologic autoimmunity) or high levels (which may indicate GAD65 neurologic autoimmunity in the appropriate clinical context). Given that cut-offs used to define “high levels” are numerically different between RIA and ELISA (e.g., 20 nmol/L by RIA vs 10,000 IU/mL by ELISA when testing serum), close attention to the assay used is essential when interpreting anti-GAD65 values. In practice, clinicians may encounter serum anti-GAD65 ELISA values of >250 IU/mL; in cases of suspected GAD65 neurologic autoimmunity the testing laboratory should be contacted to perform dilutions, which are necessary to determine whether or not the patient has a serum anti-GAD65 ELISA value in excess of 10,000 IU/mL.^76^ Importantly, high serum levels of anti-GAD65 by RIA or ELISA have been reported to have imperfect PPV for GAD65 neurologic autoimmunity, and may rarely be found in other scenarios (e.g., patients with T1DM who do not have neurologic symptoms).^75,77^ Clinical correlation of high serum anti-GAD65 levels is thus mandatory prior to diagnosing GAD65 neurologic autoimmunity. It should be noted that, much like how a high serum anti-GAD65 value alone does not confirm neurologic autoimmunity, a low serum anti-GAD65 value alone does not completely exclude this possibility^78^; the potential for a treatment-responsive autoimmune neurologic disease must therefore be considered in patients with low serum anti-GAD65 levels but clinical presentations suspicious for neurologic autoimmunity (e.g., limbic encephalitis), and additional investigations (e.g., more comprehensive panel-based neural antibody testing if not already done, like is discussed later) should be pursued as appropriate. For those with high serum anti-GAD65 levels, a typical disease phenotype (e.g., SPSD), and no concern for an alternative etiology of symptoms, lumbar puncture for confirmatory testing of anti-GAD65 in CSF may not be required for diagnosis.^71,79,80^ If there is diagnostic uncertainty (e.g., in those with equivocal disease phenotypes), then testing of CSF alongside serum to assess for intrathecal synthesis of anti-GAD65 has been suggested,^81^ although further evidence to support this recommendation is needed.

## Neural antibodies in AE: When and what to test

While neural antibody testing is a cornerstone of AE diagnosis, its role in disease monitoring is less established. Correlation of antibody titers with disease activity is imperfect and antibody positivity may persist after clinical recovery^58^; for these reasons the role of repeat antibody testing to monitor disease activity, especially in patients who are doing well clinically, is unclear. For some antibodies, demonstration of seronegativity after 6–12 months has been associated with lower rate of relapse, but the substantial proportion of persistently seropositive patients who similarly do not relapse makes it challenging to determine how to best apply this finding in clinical practice.^57,82^ Given the uncertainty surrounding repeat neural antibody testing to monitor disease activity or predict disease relapse, we herein focus on the more established role of initial neural antibody testing to diagnose AE.

Samples submitted for neural antibody testing should ideally be collected acutely at time of attack and prior initiation of immunotherapy, because both the passage of time as well as the use immunotherapies can adversely impact the diagnostic test performance.^57^ False-negatives are possible following immunotherapy and are of particular concern in patients who receive PLEX or neonatal Fc receptor inhibitors, which can rapidly reduce serum IgG levels by >70%.^83–86^ Meanwhile, false-positives may also occur if testing is performed after administration of IVIG. As an example, anti-GAD65 has been detected in IVIG preparations using ELISA; however, anti-GAD65 levels are relatively low (e.g., <2000 IU/mL by ELISA) and thus should not cause diagnostic confusion if appropriately interpreted in a patient who has undergone testing shortly after receiving IVIG.^87^ In such scenarios, repeat testing in 3–6 months can help confirm the presence of a transiently elevated antibody result secondary to IVIG.^88^ Importantly, highly specific antibody results (e.g., with positivity observed by LB/CBA alongside TBA) cannot generally be attributed to IVIG; for this reason, in patients for whom there is diagnostic uncertainty surrounding an antibody result of potential clinical relevance, reviewing details of testing that can inform specificity (discussed earlier) is crucial.

With respect to which fluid to test, submission of both serum and CSF for neural antibody testing is generally recommended in patients with suspected AE.^1,3,19^ In order to maintain antibody stability, serum and CSF samples should be promptly refrigerated at ~4°C (if able to arrange testing in the short-term) or frozen at ⩽−20°C (if there are anticipated testing delays) and multiple freeze-thaw cycles should be avoided.^89^ At the authors’ laboratory, it is recommended that clinical service basis testing of samples be performed within 14 days if refrigerated or 30 days if frozen, with the aim of minimizing time between sample collection and testing. The recommendation to submit both serum and CSF stems from the fact that some antibodies are more sensitively detected in serum using commercially available assays (e.g., anti-LGI1, CASPR2), while others are more sensitively and specifically detected in CSF (e.g., anti-NMDAR, anti-glial fibrillary acidic protein).^1,10,19,58,90,91^ Scrutiny of patients for whom certain antibodies are detected in serum only is prudent; this has been best demonstrated with anti-NMDAR, for which negative testing of accompanying CSF is a red flag for a false-positive serum result.^47,92^ For numerous rarer neural antibodies, the preferred fluid to test is not well established, further supporting submission of both serum and CSF.^10^ Because of substantial syndromic overlap of many neural antibodies associated with AE, comprehensive panel-based testing of serum and CSF serves to maximize sensitivity of testing that may be time-sensitive (i.e., because prompt confirmation of antibody-associated AE informs immunotherapy choice, cancer screening, and prognosis), whereas sequential single antibody testing can result in diagnostic delay.^1,10,19,93^ Submission of adequate serum and CSF volume is necessary to perform comprehensive neural antibody panels that may incorporate multiple assays (e.g., TBA, CBA, IB, ELISA); the volume of serum and CSF requested by the testing laboratory should therefore be reviewed prior to sample collection, and in the authors’ experience can range from 2 to 5 mL. Panel-based testing also permits the identification of multiple antibodies that can be missed if only single antibody testing is performed, which may have clinical implications (e.g., missed clue suggesting the need for more intensive cancer screening in patients with multiple antibodies associated with a particular tumor). There are unique scenarios in which single antibody testing may be sufficient (e.g., the presence of faciobrachial dystonic seizures in a patient with limbic encephalitis, which is virtually pathognomonic for anti-LGI1)^94^; however, in patients presenting more generically with the syndrome of encephalitis, panel-based testing of serum and CSF minimizes the risk of missing potentially clinically relevant antibodies. Importantly, certain antibodies may not be included in some panels, including those with relatively discrete presentations (e.g., acute disseminated encephalomyelitis or cerebral cortical encephalitis/FLAIR-hyperintense lesions in anti-MOG-associated encephalitis with seizures with anti-MOG^95,96^; SPSD including PERM with anti-GlyR, which may occasionally be detected alongside high serum levels of anti-GAD65^68,97,98^). If one is particularly concerned about neural antibody-associated AE in a patient with negative test results, then reviewing test comprehensiveness with the laboratory is recommended.

## Conclusion

The detection of neural antibodies in AE has become increasingly central to patient diagnosis, treatment, and prognostication in recent years. This trend is likely to continue as data emerge from treatment trials in antibody-mediated encephalitis that are currently underway. In parallel to the growing importance of these antibodies in patient management, commercialization of assays used for their detection has resulted in a dramatic rise in access to this testing worldwide. While improved availability of diagnostic testing has clear benefits to clinicians caring for these patients, knowledge of the strengths as well as the limitations of this testing is crucial to avoid misdiagnosis due to false-negative or false-positive results. Through review of the “who, what, where, when, why, and how” of neural antibody testing in AE, we aim to optimize the use of this testing in clinical practice.

## Declarations

**Ethics approval and consent to participate:** Not applicable.

**Consent for publication:** Not applicable.

**Availability of data and materials:** Not applicable.
